# A phasor‐based approach to improve optical sectioning in any confocal microscope with a tunable pinhole

**DOI:** 10.1002/jemt.24178

**Published:** 2022-06-10

**Authors:** Morgana D'Amico, Elisabetta Di Franco, Elena Cerutti, Vincenza Barresi, Daniele Condorelli, Alberto Diaspro, Luca Lanzanò

**Affiliations:** ^1^ Department of Physics and Astronomy “Ettore Majorana” University of Catania Catania Italy; ^2^ Nanoscopy, CHT Erzelli Istituto Italiano di Tecnologia Genoa Italy; ^3^ Department of Biomedical and Biotechnological Sciences, Section of Medical Biochemistry University of Catania Catania Italy; ^4^ DIFILAB, Department of Physics University of Genoa Genoa Italy

**Keywords:** confocal microscopy, optical sectioning, phasors, pinhole, separation of photons by lifetime tuning

## Abstract

Confocal fluorescence microscopy is a well‐established imaging technique capable of generating thin optical sections of biological specimens. Optical sectioning in confocal microscopy is mainly determined by the size of the pinhole, a small aperture placed in front of a point detector. In principle, imaging with a closed pinhole provides the highest degree of optical sectioning. In practice, the dramatic reduction of signal‐to‐noise ratio (SNR) at smaller pinhole sizes makes challenging the use of pinhole sizes significantly smaller than 1 Airy Unit (AU). Here, we introduce a simple method to “virtually” perform confocal imaging at smaller pinhole sizes without the dramatic reduction of SNR. The method is based on the sequential acquisition of multiple confocal images acquired at different pinhole aperture sizes and image processing based on a phasor analysis. The implementation is conceptually similar to separation of photons by lifetime tuning (SPLIT), a technique that exploits the phasor analysis to achieve super‐resolution, and for this reason we call this method SPLIT‐pinhole (SPLIT‐PIN). We show with simulated data that the SPLIT‐PIN image can provide improved optical sectioning (i.e., virtually smaller pinhole size) but better SNR with respect to an image obtained with closed pinhole. For instance, two images acquired at 2 and 1 AU can be combined to obtain a SPLIT‐PIN image with a virtual pinhole size of 0.2 AU but with better SNR. As an example of application to biological imaging, we show that SPLIT‐PIN improves confocal imaging of the apical membrane in an in vitro model of the intestinal epithelium.

**Research Highlights:**

We describe a method to boost the optical sectioning power of any confocal microscope. The method is based on the sequential acquisition of multiple confocal images acquired at different pinhole aperture sizes. The resulting image series is analyzed using the phasor‐based separation of photons by lifetime tuning (SPLIT) algorithm. The SPLIT‐pinhole (SPLIT‐PIN) method produces images with improved optical sectioning but preserved SNR. This is the first time that the phasor analysis and SPLIT algorithms are used to exploit the spatial information encoded in a tunable pinhole size and to improve optical sectioning of the confocal microscope.

## Graphical abstract

Schematic of the SPLIT‐PIN method: a stack of confocal images acquired with tunable pinhole size is processed by phasor analysis to produce a final image without out‐of‐focus background.

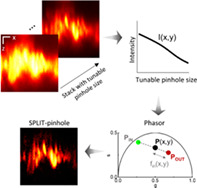

## 1 INTRODUCTION

Confocal fluorescence microscopy is a very popular and versatile tool in life sciences (Conchello & Lichtman, 2005; Jonkman & Brown, 2015). Compared to wide‐field fluorescence microscopy, the main advantage of confocal microscopy is represented by its ability to remove the unwanted blur originating from out‐of‐focus planes and generate thin optical sections of the specimen. By scanning along the optical axis, confocal microscopy can provide three‐dimensional reconstructions of biological specimens. An important feature of point‐scanning confocal microscopy is that it can be easily combined with many modern quantitative fluorescence techniques. Confocal‐based fluorescence lifetime imaging (FLIM) is a powerful technique for the detection of forster resonance energy transfer (FRET) (Broussard et al., 2013; Day, 2014; Giral et al., 2011; Pelicci et al., 2019) and the imaging of fluorescent sensors (Ferri et al., 2016; Scipioni et al., 2021). Confocal‐based spectral imaging can be used to separate multiple spectral components (Fereidouni et al., 2012; Shi et al., 2020) and to analyze the response of environment‐sensitive fluorescent dyes (Malacrida et al., 2016; Sediqi et al., 2018). Confocal‐based fluorescence recovery after photobleaching (FRAP) and fluorescence correlation spectroscopy (FCS) are well‐established methods to measure the diffusion of molecules inside live cells (Di Bona et al., 2019; Fritzsche & Charras, 2015; Scipioni et al., 2018). Notably, stimulated emission depletion microscopy (STED), one of the most popular super‐resolution imaging techniques, is also based on a confocal microscope architecture (Vicidomini et al., 2018).

In confocal microscopy, optical sectioning is provided by the pinhole, a small aperture placed in front of the point detector, whose position is confocal to the illuminated spot in the specimen (Diaspro, 2019; Pawley, 2006). The size of the pinhole directly determines the degree of optical sectioning. The use of smaller pinhole sizes improves the discrimination of in‐focus light versus out‐of‐focus light. In addition, imaging with smaller pinholes also improves the lateral resolution with a maximum theoretical improvement of a factor of 1.4 for a fully closed pinhole (Conchello & Lichtman, 2005; Diaspro, 2019). Unfortunately, the reduction of the pinhole size drastically reduces light throughput at the detector (reduction of about 95% of the intensity from 1 Airy Unit (AU) to 0.2 AU (Huff, 2015) so that one has to find a compromise between optical sectioning and signal‐to‐noise ratio (SNR) (Sheppard et al., 2006). The reduction of SNR at smaller pinhole sizes makes difficult, in practice, the use of pinhole sizes significantly smaller than 1 AU.

Here, we propose a simple approach to “virtually” perform confocal imaging at smaller pinhole sizes without the dramatic reduction of SNR. The approach is based on the concept of separation of photons by lifetime tuning (SPLIT) (Lanzano et al., 2015), a super‐resolution technique originally introduced in the context of lifetime‐resolved STED microscopy. In SPLIT, the increase in spatial resolution is obtained by decoding the spatial information encoded into the lifetime channel of the microscope. In this additional channel, the signal originating from the center of the excitation spot has a different fingerprint (longer lifetime) compared to the signal coming from the periphery of the excitation spot (shorter lifetime). This fingerprint can be exploited to isolate, via phasor analysis, the fraction of the signal originating from the center of the excitation spot and generate a super‐resolved image (Lanzano et al., 2015). We have recently demonstrated that the SPLIT approach to super‐resolution is not limited to lifetime‐resolved images [Sarmento 2018] but has a more general applicability. In other words, the same phasor algorithm can be applied to microscopy images containing an additional channel with encoded spatial information. In STED microscopy, this additional channel can be represented by the fluorescence lifetime variations induced by a STED beam (Coto Hernandez et al., 2019; Lanzano et al., 2015; Lanzano et al., 2017; Tortarolo et al., 2019; Wang et al., 2018) or a tunable depletion power (Pelicci et al., 2020; Sarmento et al., 2018). In STED microscopy, the application of SPLIT produces images that have higher resolution and better contrast, compared to their counterpart STED images (Cerutti et al., 2021). Recently, we have shown that SPLIT can be applied also to structured illumination microscopy (SIM), using as the additional channel the illumination pattern translation step (Cainero et al., 2021). In confocal microscopy, the tunable pinhole size encodes spatial information in the axial direction. In fact, for decreasing values of the pinhole size, the percentage contribution of the out‐of‐focus intensity to the total intensity decreases, resulting in a different fingerprint between the out‐of‐focus and in‐focus intensity components. Thus, the tunable pinhole size can be used as the additional channel for application of the phasor‐based SPLIT algorithm.

The SPLIT‐pinhole (SPLIT‐PIN) method is based on the sequential acquisition of multiple confocal images acquired with a different pinhole size. The images are processed to obtain a final image with improved optical sectioning (i.e., virtually smaller pinhole size) but preserved SNR level. Notably, our method can be implemented on any confocal microscope equipped with a tunable pinhole size (to the best of our knowledge, most of the commercially available confocal laser scanning microscopes allow the users to tune the size of the pinhole). To evaluate the quality of the images provided by SPLIT‐PIN, we use the recently introduced QuICS algorithm, a tool based on image correlation spectroscopy (Cerutti et al., 2021). QuICS allows extracting three parameters related to the resolution, contrast e SNR of the image. We show with simulations that the SPLIT‐PIN image can provide improved optical sectioning (i.e. virtually smaller pinhole size) with respect to confocal images but better SNR with respect to an image obtained with closed pinhole. For instance, two images acquired at 2 AU and 1 AU can be combined to obtain a SPLIT‐PIN image with a virtual pinhole size of 0.2 AU but with better SNR. As an example of application to biological imaging, we apply SPLIT‐PIN to confocal imaging of the apical membrane in an in vitro model of the intestinal epithelium and we find that the SPLIT‐PIN image has a better contrast compared to conventional confocal imaging. In summary, we demonstrate that SPLIT‐PIN is can be a simple and effective tool to boost the optical sectioning power of any confocal microscope.

## 2 MATERIALS AND METHODS

### 2.1 Cell culture and labeling

Human colon cancer Caco‐2 (ATCC number: HTB‐37) cell line was maintained as the previously described (Barresi et al., 2016) in Dulbecco's Modified Eagle Medium (DMEM; GIBCO, Cat No. 31965–023 containing 4.5 g/L^−1^ of D‐glucose) supplemented with 20% FBS (Cat. No. 10270–106; Life Technologies, Monza, Italy) and 100 U/mL ^−1^ of penicillin–streptomycin (Cat. No 15140–122; Life Technologies). The cell culture was grown in flasks (25 cm^2^) and incubated at 37°C in humidified atmosphere with 5% of CO_2_ and 95% of air. The culture medium was changed twice a week. Cells were seeded on 8‐well chambered coverslips (μ‐Slide 8 Well Glass Bottom, ibidi 80827, Germany) and let growing until 100% confluence. Then, cells were stained with CellMask Orange (Thermofisher C10045) at a dilution 1:1000, for 10 min at 37°C. Next, cells were washed twice with PBS and fixed with 4% PFA, at room temperature for 10 min. Finally, cells were washed with PBS and covered with ProLong Diamond Antifade Mountant.

### 2.2 Image acquisition

Images were acquired on a Leica TCS SP8 confocal microscope, using an HCX PL APO CS2 63X 1.40 NA oil immersion objective lens (Leica Microsystems, Mannheim, Germany). Tetraspeck fluorescent spheres with a size of 200 nm (TetraSpeck Fluorescent Microspheres Size Kit, ThermoFisher) were excited at 488 nm and fluorescence emission detected at 500–550 nm. CellMask Orange was excited at 561 nm and its fluorescence emission detected at 565–650 nm using a hybrid detector (Leica Microsystems). Series of multiple confocal images at different pinhole sizes were acquired using the frame‐sequential acquisition. The pinhole size was set as specified. The excitation power was kept constant unless specified otherwise. The number of line averaging was kept constant unless specified otherwise.

### 2.3 Simulations

Image stacks representing confocal *xz* sections acquired with a tunable pinhole size were simulated using MATLAB. The objects consisted in a variable number of point‐like emitters distributed randomly in a matrix of 256×256 with a pixel size of 50nm. To generate the stack, the object was convolved with a Point Spread Function (PSF) given by PSF(*x*,*z*,*s*) = PSF(*x*)PSF(*z*,*s*), where PSF(*x*) and PSF(*z*,*s*) are Gaussian functions and *s* represents the pinhole size. For simplicity, we simulated a variation with the pinhole size only for the PSF along the *z* axis. We set PSF(*x*) = exp(−2*x* ^2^/*w*_*x*_^2^), with *w*_*x*_ = 169 nm, whereas PSF(*z*,*s*) was given by

(1) $$\mathrm{PSF}\left(z,s\right)=A\left(s\right)\exp\left(-2z^{2}/{w_{z}}^{2}\left(s\right)\right),$$

where *w*_z_(*s*) is the 1/*e* ^2^ width of the PSF, represented as a function of the pinhole size *s*, and *A*(*s*) is an amplitude factor that takes into account the decrease of the transmitted intensity through the pinhole. Specifically, we simulated a stack *F*_*j*_(*x*,*y*), with *j* = 1, …, *n* (with *n* = 3), representing an acquisition with decreasing pinhole size (size 1.5 A.U., 1 A.U., 0.5 A.U). The stack consisted of three images with decreasing waist along z, w_z_(s), given by 680, 620, and 590 nm, and a decreasing amplitude A(s) given by 1.4 S, S, and 0.38 S, where S represents the maximum number of photon counts detected for a single object in the frame corresponding to 1 A.U.

For comparison, we simulated a single image representing an acquisition with a fixed pinhole size (size 0.2 A.U.) but with 3‐times more signal. The single frame image was simulated with a waist along *z* corresponding to 550 nm and number of counts given by 3 × 0.1 S = 0.3 S.

The specific values of *w*(*s*) and *A*(*s*) used in the simulations were determined so as to correspond to the parameters of the axial PSF detected on our confocal setup. In the simulations, the total number of photons detected was varied and three different values of S were used, that is, *S* = 50, 100, and 500. For each condition, the evaluation was performed on five replicates.

### 2.4 Phasor plot and generation of SPLIT‐PIN images

For a given image stack $F_{j}\left(x,y\right)$, the images were processed with the phasor analysis in which variables $g\left(x,y\right)$ and $s\left(x,y\right)$ were calculated as(Digman et al., 2008; Malacrida et al., 2021):

(2) $$g\left(x,y\right)=\frac{\sum_{j=1\;}^{n}F_{j}\left(x,y\right)\cos\left[\frac{2\pi \left(j-1\right)}{n}\right]}{\sum_{j=1\;}^{n}F_{j}\left(x,y\right)},$$

(3) $$s\left(x,y\right)=\frac{\sum_{j=1\;}^{n}F_{j}\left(x,y\right)\sin\left[\frac{2\pi \left(j-1\right)}{n}\right]}{\sum_{j=1\;}^{n}F_{j}\left(x,y\right)},$$

where *n* is the number of the images of the stack.

The modulation $M\left(x,y\right)$ and the phase $\phi \left(x,y\right)$ are the polar coordinates of the phasor and were calculated as follows:

(4) $$M\left(x,y\right)={\left(g^{2}\left(x,y\right)+s^{2}\left(x,y\right)\right)}^{1/2},$$

(5) $$\phi \left(x,y\right)=\tan^{-1}\frac{s\left(x,y\right)}{g\left(x,y\right)}.$$

For each pixel, the fraction $f_{\text{in}}\left(x,y\right)$ of fluorescence intensity associated with the center of the PSF was calculated by expressing the experimental phasor $P\left(x,y\right)$ as a combination of the phasors **P**_IN_ and **P**_OUT_ which represent, respectively, the center and the periphery of the PSF and can be determined directly in the phasor plot. This fraction was estimated as follows:

(6) $$f_{\text{in}}=\left(P_{\text{OUT}}-P\left(x,y\right)\right)∙\left(P_{\text{OUT}}-P_{\text{IN}}\right)/{\left|P_{\text{OUT}}-P_{\text{IN}}\right|}^{2},$$

that is, proportional to the distance between the phasor P and the phasor $P_{\text{OUT}}$ along the line connecting $P_{\text{IN}}$ and $P_{\text{OUT}}$. In the analysis of simulated data, P_IN_ and P_OUT_ were set as *P*_IN_ = (0.25, 0.19) and *P*_OUT_ = (0.35, 0.19). To force values of fraction to fall between 0 and 1, the values of *f*_IN_(*x*, *y*) were filtered through a logistic function of the form $f=1/\left(1+e^{-k_{L}\left(f-1/2\right)}\right)$, with *k*_L_ = 4(Cainero et al., 2021).

Finally, the SPLIT‐PIN image was calculated as follows:

(7) $$I_{\text{SPLIT}-\mathrm{PIN}}\left(x,y\right)=f_{\text{IN}}\left(x,y\right)I_{\text{conf}}\left(x,y\right),$$

where *I*_conf_ can be any of the confocal images *F*_*j*_(*x*,*y*) or their sum.

### 2.5 Image analysis

The generated SPLIT‐PIN images were analyzed using a recently introduced algorithm that evaluates the image quality by image correlation spectroscopy (QuICS) (Cerutti et al., 2021). The QuICS analysis was performed in MATLAB using the code QuICS_v2.m available at https://github.com/llanzano/QuICS. The algorithm was modified to include the possibility to calculate the image autocorrelation function separately along the x and y direction of the image. Briefly, given an image *I*(*x*,*y*), a two‐dimensional (2D) image correlation function *G*_2D_(*δ*_x_,*δ*_*y*_) was calculated as follows:

(8) $$G_{2D}\left(\delta_{x},\delta_{y}\right)=\frac{\left\langle I\left(x,y\right)I\left(x+\delta_{x},y+\delta_{y}\right)\right\rangle }{{\left\langle I\left(x,y\right)\right\rangle }^{2}}-1$$

where *δ*_*x*_ and *δ*_*y*_ are the spatial lag variables, *I*(*x*,*y*) is the fluorescence intensity detected at pixel, and (*x*,*y*) is the angle brackets indicate averaging over all the selected pixels of the image. The numerator in Equation (8) was calculated by a 2D fast Fourier transform algorithm. The autocorrelation function along the *x* axis, *G*_*x*_, was calculated as *G*_*x*_(*δ*_*x*_) = *G*_2D_(*δ*_*x*_,0). The autocorrelation function along the *y* axis, *G*_*y*_, was calculated as *G*_*y*_(*δ*_*y*_) = *G*_2D_(0, *δ*_*y*_) (Note: for the analysis reported in Figure 3, the *y* axis of the image corresponds to the *z* axis of the microscope). The radial correlation function *G*(*δ*_*r*_) was calculated by performing an angular mean (Scipioni et al., 2016).

The noise‐free correlation function was estimated by performing a Gaussian fit of the correlation function G(*δ*) by skipping the zero lag point:

(9) $$\begin{array}{c} G_{\mathrm{NF}}\left(\delta \right)=G_{\mathrm{NF}}\left(0\right)e^{-\frac{\delta^{2}}{w^{2}}}+G_{\mathrm{NF}}\left(\infty \right)\\ \delta \in \left[1,\delta_{\max}\right] \end{array},$$

where the width parameter *w* corresponds to the 1/e^2^ of a Gaussian function and it is related to the full‐width half maximum (FWHM) by the relationship w = FWHM/(2ln2)^1/2^; *G*_NF_(0) represents the amplitude; *G*_NF_(∞) represents an offset value. The value δ_max_ was set in such a way to fit a single Gaussian component. Finally, the parameters *R*, *B*, and *N* have been calculated as follows:

(10) $$\begin{array}{c} R=\sqrt{2\ln2}w,\\ B=G_{\mathrm{NF}}\left(0\right)I_{\mathrm{av},}\\ N=\frac{G\left(0\right)-G_{\mathrm{NF}}\left(0\right)}{G_{\mathrm{NF}}\left(0\right)}, \end{array}$$

where we have indicated *I*_av_ as the average intensity value over all the pixels of the image. *R* is the width of the autocorrelation function, related to the spatial resolution; *B* is the brightness, related to the image contrast; and *N* is the relative noise variance, related to the SNR of the image (Cerutti et al., 2021).

## 3 RESULTS

### 3.1 Spatial information is encoded in a tunable pinhole size

**FIGURE 1:**
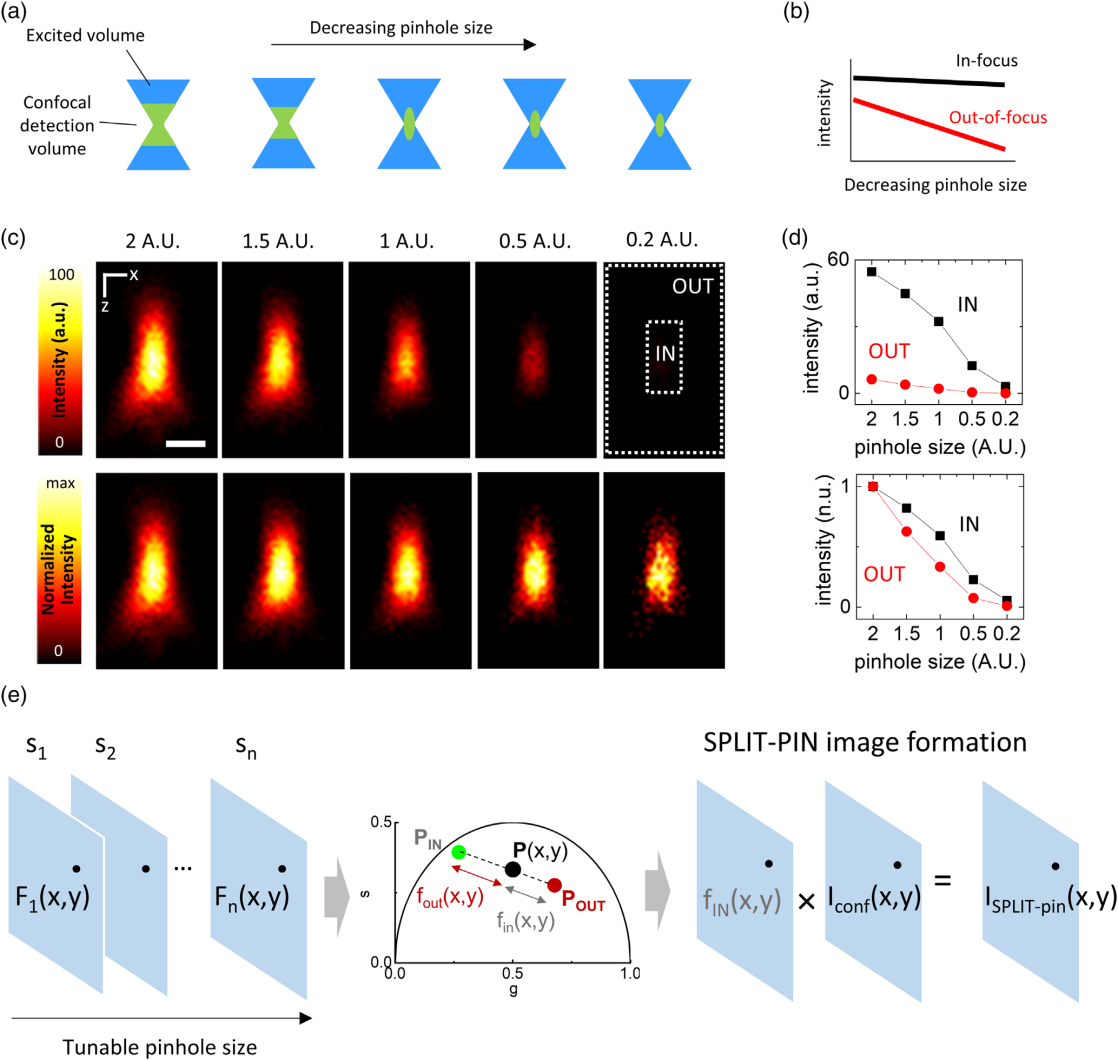
Encoding of spatial information in confocal microscopy via a tunable pinhole size. (a) Schematic showing the effect of a decreasing pinhole size on the detection volume of a confocal microscope. (b) Schematic showing the variation of the in‐focus (black) and out‐of‐focus (red) intensity as a function of a decreasing pinhole size. (c) Confocal *xz* images of 200‐nm fluorescent beads acquired at diferent pinhole sizes, expressed in Airy Units (A.U.) Shown is the intensity (top) or the intensity normalized to the maximum (bottom). Scale bar represents 500 nm. (d) Average intensity in the IN and OUT ROIs defined in (c) as a function of the pinhole size. Shown are the intensity (top) and the intensity normalized to the value at 2 A.U. (bottom). (e) Schematic workflow of the SPLIT‐PIN method. (from left to right) A series of n images is acquired with a tunable pinhole size; for each pixel, the phasor *P*(*x,y*) is calculated from the intensity as a function of the frame index in the series; the fraction *f*_IN_(*x*,*y*) is calculated by decomposition of the phasor P(x,y) in two components; the SPLIT‐PIN image is calculated as the product of *f*_IN_(*x*,*y*) and a confocal image.

In confocal microscopy, it is the size of the pinhole aperture that determines the extent of optical sectioning. For decreasing values of the pinhole size, an increasing amount of out‐of‐focus light is rejected (Figure 1a). Thus, for decreasing values of pinhole size, the intensity originating from the out‐of‐focus part of the excitation volume decays more rapidly than the intensity originating from the in‐focus part of the same excitation volume (Figure 1b). We can consider the tunable pinhole size as an additional microscope channel. Along this channel, the out‐of‐focus component and the in‐focus component are distinguishable because they have a different fingerprint (Figure 1b). Approximating the confocal point spread function (PSF) along the *z* axis as a Gaussian function, see Equation (1), the width w_z_(s) decreases as a function of the pinhole size *s*, and *A*(*s*), an amplitude factor that represents the transmitted intensity through the pinhole, also decreases. For a fluorophore in focus (*z* = 0):

PSF(0,*s*) = *A*(*s*) (11)

for a fluorophore located out‐of‐focus (z_0_ > 0).

PSF(*z*_0_,*s*) = *A*(*s*)exp(−2*z*_0_^2^/*w*_*z*_^2^(*s*)) (12)

Figure 1c shows confocal *xz* images of 200‐nm fluorescent spheres using decreasing values of pinhole size. The images in Figure 1c have been obtained sequentially using the same excitation power and the same number of line‐averages. As expected, when the pinhole size is changed from 2 Airy Units (A.U.) down to 0.2 A.U., we observe an improvement of the optical sectioning but also a significant decrease of the signal level. By defining a ROI that correspond to an in‐focus region (IN) and a ROI that corresponds to an out‐of‐focus region (OUT), we can observe that the IN and OUT components of the fluorescence signal have a different fingerprint as a function of the tunable pinhole size (Figure 1d). The OUT component decays faster than the IN component. Thus, the tunable pinhole size s can be used as an additional channel of the microscope that encodes spatial information.

This information can be visualized using phasor analysis (Digman et al., 2008; Malacrida et al., 2021) along the dimension s (Figure 1e). In the phasor plot, we can decompose the phasor of each pixel into the phasor of the IN component (*P*_IN_) and the phasor of the OUT component (P_OUT_), to obtain the fraction *f*_IN_(*x*,*y*) of the intensity corresponding to the in‐focus region of the PSF. This fraction can multiplied by any of the available confocal images (or any sum of the available confocal images) *I*_conf_ to obtain the final SPLIT‐PIN image.

### 3.2 Advantage of SPLIT‐PIN versus closed pinhole imaging

**FIGURE 2:**
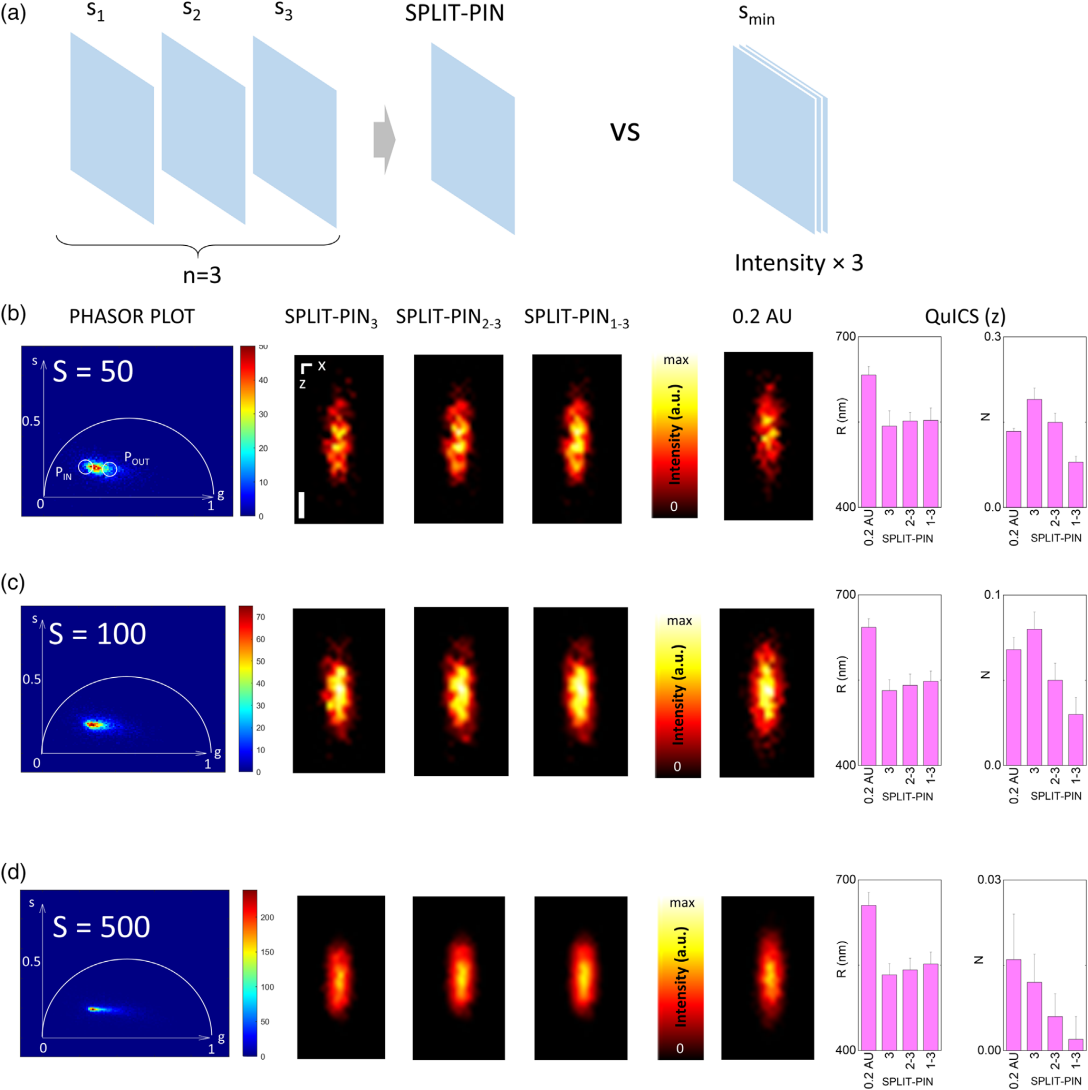
Characterization of the SPLIT‐PIN image by simulations. (a) Schematic of the simulated data used for evaluating the advantage of using a tunable pinhole size. (b–d) Analysis of the simulated data for different levels of simulated photon counts: *S* = 50 (b), *S* = 100 (c), and *S* = 500 (d). Shown are (from left to right) the phasor plot, the three SPLIT‐PIN images obtained from the simulated stack, the simulated image at 0.2 A.U., the evaluation of resolution along *z* (*R*) and the noise level (*N*) by the QuICS algorithm. Scale bar 300 nm.

Here, we evaluate through simulated data the advantage in performing an acquisition with tunable pinhole size plus image processing (SPLIT‐PIN) versus an acquisition with a closed pinhole. We simulated an acquisition with 3 different pinhole sizes (1.5, 1, and 0.5 AU) and compared the resulting SPLIT‐PIN image with a single frame acquired at 0.2 AU with a comparable integration time (Figure 2a). For the comparison, we evaluated three SPLIT‐PIN images generated multiplying the fraction f_IN_(x,y) by the last image of the stack (SPLIT‐PIN_3_), the sum of frame 2 and 3 of the stack (SPLIT‐PIN_2‐3_), the sum of the frames 1 to 3 of the stack (SPLIT‐PIN_1‐3_).

To evaluate quantitatively the improvement provided by the SPLIT‐PIN approach, we applied the recently developed QuICS algorithm (Cerutti et al., 2021) to the images. QuICS exploits the calculation of a radial (i.e., angularly averaged) spatial autocorrelation function (ACF) to extract three parameters related to the quality of the image. Here, given the asymmetry of the *xz*‐image, we modified the algorithm in order to calculate an ACF along the x direction and an ACF along the *z* direction, instead of a single radial ACF. QuICS along the z axis reveals that, in all the cases, the SPLIT‐PIN images (SPLIT‐PIN_3_, SPLIT‐PIN_2‐3_, and SPLIT‐PIN_1‐3_) have better resolution (parameter R representing the resolution expressed as full‐width at half maximum (FWHM)) than the image at 0.2 A.U (Figure 2b–d). The noise level of the SPLIT‐PIN images (parameter N of the QuICS analysis) depends on the simulated photon counts S and vary between SPLIT‐PIN_3_, SPLIT‐PIN_2‐3_, SPLIT‐PIN_1‐3_, and being lowest for SPLIT‐PIN_1‐3_. In all cases, the noise level of SPLIT‐PIN_1‐3_ is lower than the noise level of the image at 0.2 A.U (Figure 2b–d).

These results indicate that, at least for the conditions of our simulations, tuning the pinhole size and generating a SPLIT‐PIN image can provide some advantages with respect to closing the pinhole and acquiring a single image. In particular, in the conditions of our simulations, the SPLIT‐PIN image has a better resolution along z (FWHM_z_—550 nm) compared to the image at 0.2 AU (FWHM_z_—650 nm). Moreover, the simulations indicate that, in the generation of the SPLIT‐PIN image, using the sum of the confocal images of the stack yields the SPLIT‐PIN image with the highest SNR, as expected.

### 3.3 SPLIT‐PIN imaging of subcellular structures

As a proof‐of‐principle of the applicability of the SPLIT‐PIN method to biological imaging, we performed imaging on fixed cells labeled with fluorescent dyes. Organic dyes are relatively small molecules compared to primary and secondary antibodies. Staining with organic dyes can reach, in general, a higher density of labeling compared to labeling with probes of larger molecular size such as the combination of primary and secondary antibodies. This higher density of labeling is often associated with the occurrence of significant out‐of‐focus background, even in relatively thin samples such as cultured cells.

We show images of CaCo‐2 cells labeled with the membrane marker CellMask Orange. CaCo‐2 cells are an in vitro model of the small intestine and have been used as a model of enterocyte transport function and regulation (Giral et al., 2012). In particular, fully confluent CaCo‐2 cells exhibit the typical epithelial cell polarity characterized by the presence of a dense array of microvilli on the apical membrane (Giral et al., 2012). Apical microvilli are actin‐based protrusions of cylindrical shape, with a length of few micrometers and a diameter of about 100 nm. High‐resolution imaging of microvilli, especially in live cells, is quite challenging and has prompted the development of several dedicated microscopy approaches (Blaine et al., 2009; Lanzano et al., 2011; Maraspini et al., 2019; Ranjit et al., 2021). In contrast, the basal membrane and the basolateral membrane have a very different morphology compared to the apical compartment. In this epithelial model, the main axis of cell polarity is along the optical axis, thus optical sectioning is fundamental to distinguish the different functional compartments of the cell. For this reason, this system represents an ideal test to evaluate the improving of the optical sectioning performances provided by the SPLIT‐PIN method.

**FIGURE 3:**
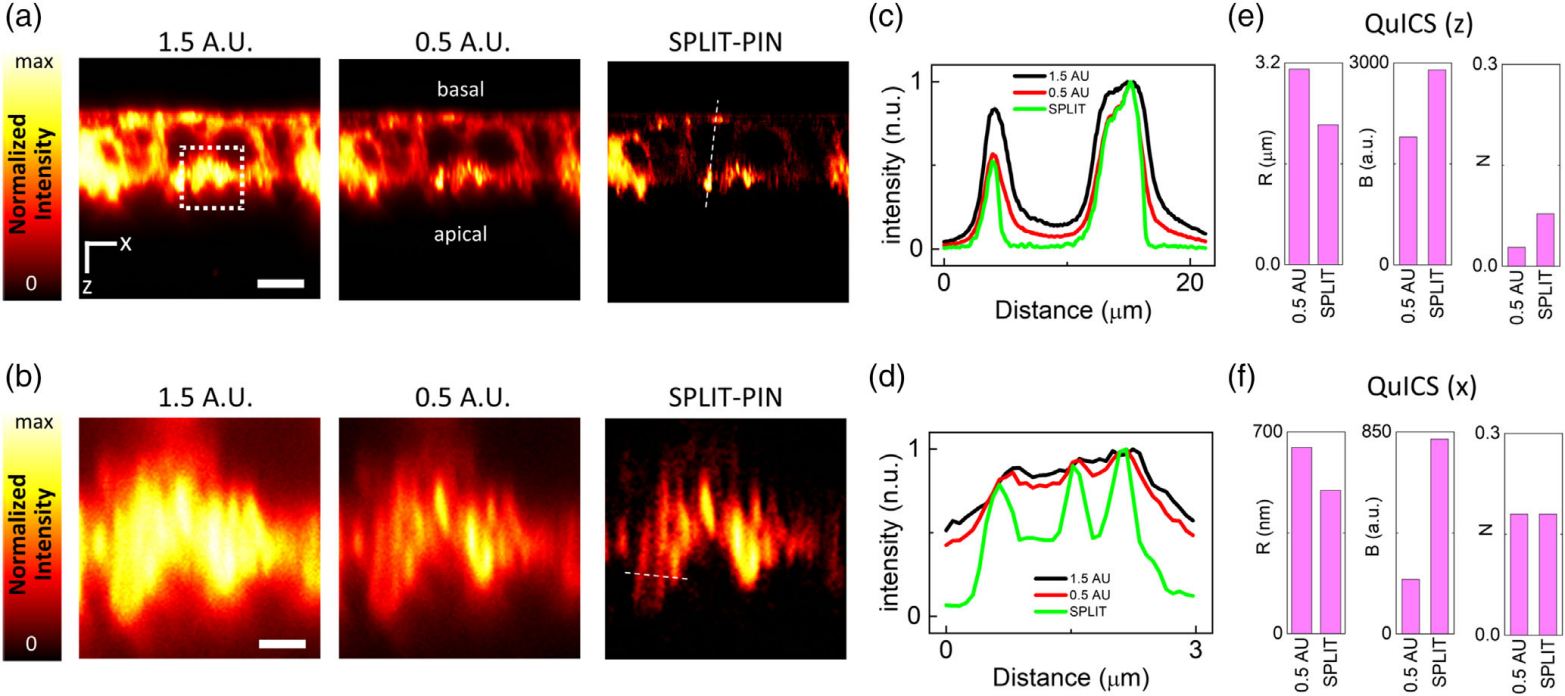
SPLIT‐PIN imaging of membranes in polarized epithelial cells. (a,b) SPLIT‐PIN imaging of fixed CaCo‐2 cells labeled with CellMask Orange. Shown are confocal images acquired at 1.5 A.U. and 0.5 A.U. and the SPLIT‐PIN image. The position of the basal and apical membranes are indicated in (a). The image in (b) shows a portion of the apical membrane. Scale bars 8 μm (a) and 2 μm (b). (c) Line profiles corresponding to the dashed line in (a). (d) Line profile corresponding to the dashed line in (b). (e,f) Quantification of Resolution (*R*), Brightness (*B*), and Noise (*N*) parameters for the SPLIT‐PIN and 0.5 A.U. images shown in (b) using the QuICS algorithm. QuICS is applied separately along the *z* axis (e) and the *x* axis (f).

Figure 3a,b shows a series of two images acquired with 1.5 and 0.5 A.U pinhole size and the corresponding SPLIT‐PIN image. SPLIT‐PIN provides a better discrimination between the apical and basal membrane compartments, as shown by a line profile across the two membranes (Figure 3c). In the apical membrane, microvilli are visualized more clearly in the SPLIT‐PIN image than in the 0.5 A.U. image (Figure 3d). To evaluate more quantitatively, the improvement provided by the SPLIT‐PIN approach, we applied the recently developed QuICS algorithm (Cerutti et al., 2021) to the images. QuICS exploits the calculation of a radial (i.e., angularly averaged) spatial autocorrelation function (ACF) to extract three parameters related to the quality of the image. Here, given the asymmetry of the apical membrane in the *xz*‐image, we slightly modified the algorithm in order to calculate an ACF along the *x* direction and an ACF along the *z* direction, instead of a single radial ACF. QuICS along the *z* axis reveals that the SPLIT‐PIN image has better resolution (*R* = 2.22 μm), higher contrast (*B* = 2900) and higher noise level (*N* = 0.078) compared to the confocal image acquired at 0.5 A.U (*R* = 3.10 μm, *B* = 1900, and *N* = 0.028). QuICS along the x axis reveals that the SPLIT‐PIN image has better resolution (*R* = 497 nm), higher contrast (*B* = 820), and the same noise level (*N* = 0.18) compared to the confocal image acquired at 0.5 A.U (*R* = 646, *B* = 230, and *N* = 0.18).

## 4 DISCUSSION

We have demonstrated that the phasor analysis of series of confocal images acquired with a different pinhole size can be used to generate an image with improved optical sectioning (i.e. a virtually smaller pinhole). Using simulated data, we observed that tuning the pinhole size and generating a SPLIT‐PIN image can produce an image of better resolution and better SNR with respect to closing the pinhole and acquiring a single image. Thus, the SPLIT‐PIN method can overcome the main limitation of confocal imaging with closed pinhole, namely the dramatic decrease of SNR. We note that this limitation can be also overcome by image scanning microscopy (ISM), a technique originally developed by Colin Sheppard in the 1980s and recently introduced in the portfolio of commercially available imaging techniques (Gregor & Enderlein, 2019). In some ISM implementations, the combination of pinhole and point‐detector is substituted by a detector array that behaves as a system with multiple, small pinholes (Castello et al., 2019; Korobchevskaya, 2017). In these setups, the reduction of SNR at small pinhole size is compensated by the large number of detectors, whose signal is recombined into a reconstructed ISM image. Image scanning microscopy provides not only improved optical sectioning but also an improvement of lateral resolution by a factor $\sim \sqrt{2}$. Here, we have investigated only the improvement of optical sectioning provided by SPLIT‐PIN. Compared to ISM, the main advantage of SPLIT‐PIN is that it can be readily applied to any confocal microscope with a tunable pinhole size (i.e., most commercially available confocal laser scanning microscopes).

We note that the advantage of using information from differences in confocal pinhole size has long been recognized (Heintzmann et al., 2003; Kakade et al., 2015; Martinez‐Corral et al., 2003; Wang et al., 2013) and the virtual adaptable aperture system (VAAS) from Nikon (Okugawa, 2008) represents a commercial implementation based on the same principle. However, most of the proposed techniques are based on the weighted subtraction of two images. Here, we take into account, the more general condition of series of images acquired with a tunable pinhole size and we use the phasor to analyze the spatial information contained in the image series. Thus, the SPLIT‐PIN method can be applied to series formed by an arbitrary number of images. In the particular case of series made of only two images, we expect the SPLIT‐PIN method to perform similarly to a subtractive imaging approach (Supplementary Figure S1).

We also note that the information contained in series of images with tunable size could be analyzed using approaches not based on phasors. For each pixel, one could fit the intensity as a function of the pinhole size to a specific functional model and isolate a component corresponding to the in‐focus region of the PSF. For instance, in a technique called dynamic saturation optical microscopy (DSOM), the time‐dependent signal at each pixel is described as the sum of exponential decay components (Humpolickova et al., 2010). In this respect, an advantage of SPLIT‐PIN (and, more in general, of SPLIT and phasor‐based methods) is that no model is required for improving the resolution.

Notably, this work is the first demonstration that the SPLIT concept can have application also outside the superresolution field. Phasor‐based super‐resolution was originally developed in lifetime‐resolved STED microscopy (Coto Hernandez et al., 2019; Lanzano et al., 2015; Tortarolo et al., 2019; Wang et al., 2018) and later extended to other (non‐lifetime) STED configurations (Pelicci et al., 2020; Sarmento et al., 2018). Recently, we demonstrated that SPLIT can be applied also to structured illumination microscopy (SIM), using as the additional channel the illumination pattern translation step (Cainero et al., 2021). Here, we have shown that the SPLIT concept can have application in confocal microscopy, based on the tunability of one of its key optical elements, the confocal pinhole. To the best of our knowledge, this is the first time that the phasor analysis and SPLIT algorithms are used to exploit the spatial information encoded in a tunable pinhole size and to improve optical sectioning of the confocal microscope.

## AUTHOR CONTRIBUTIONS

Luca Lanzanò and Alberto Diaspro designed the study. Luca Lanzanò, Morgana D'Amico, and Elisabetta Di Franco wrote manuscript. Elena Cerutti, Morgana D'Amico, and Vincenza Barresi prepared samples. Luca Lanzanò, Elena Cerutti, Elisabetta Di Franco, and Morgana D'Amico collected data. Luca Lanzanò wrote software. Elisabetta Di Franco, Morgana D'Amico, Elena Cerutti, Vincenza Barresi, Daniele Condorelli, Alberto Diaspro, and Luca Lanzanò analyzed data and discussed results. All authors critically reviewed the manuscript.

## CONFLICT OF INTEREST

The authors declare no competing interests.

## Supporting information


**Figure S1** Comparison between subtractive imaging and SPLIT‐PIN imaging. (a) Subtractive imaging applied to the data of Figure 3. The processed image has been calculated as IPH2‐γ(IPH1‐IPH2), with *γ* = 0.15, where PH2 = 0.5 AU and PH1 = 1.5 AU. (b) The SPLIT‐PIN image is shown for comparison. Scale bars 8 μm.

## Data Availability

The data that support the findings of this study are available from the corresponding author upon reasonable request.
