# Supplementary material for: A phasor‐based approach to improve optical sectioning in any confocal microscope with a tunable pinhole
Source: Microsc Res Tech. 2022 Jun 10;85(9):3207–16. doi: 10.1002/jemt.24178 (PMC9542401; doi:10.1002/jemt.24178)
Supplement: Supplementary file 1 — Figure S1 Comparison between subtractive imaging and SPLIT‐PIN imaging. (a) Subtractive imaging applied to the data of Figure 3. The processed image has been calculated as IPH2‐γ(IPH1‐IPH2), with γ = 0.15, where PH2 = 0.5 AU and PH1 = 1.5 AU. (b) The SPLIT‐PIN image is shown for comparison. Scale bars 8 μm. [file JEMT-85-3207-s001.docx]

Supporting information for:

A phasor-based approach to improve optical sectioning in any confocal microscope with a tunable pinhole

Morgana D’Amico^1*^, Elisabetta Di Franco^1*^, Elena Cerutti^1,2*^, Vincenza Barresi^3^, Daniele Condorelli^3^, Alberto Diaspro^2,4^, Luca Lanzanò^1,2^.

^1^Department of Physics and Astronomy “Ettore Majorana”, University of Catania, Catania, Italy; ^2^Nanoscopy, CHT Erzelli, Istituto Italiano di Tecnologia, Genoa, Italy; ^3^Department of Biomedical and Biotechnological Sciences, Section of Medical Biochemistry, University of Catania, Catania, Italy; ^4^DIFILAB, Department of Physics, University of Genoa, Genoa, Italy.

^*^Equal contribution

Corresponding author: [luca.lanzano@unict.it](mailto:luca.lanzano@unict.it)

Content: Supplementary Fig.S1


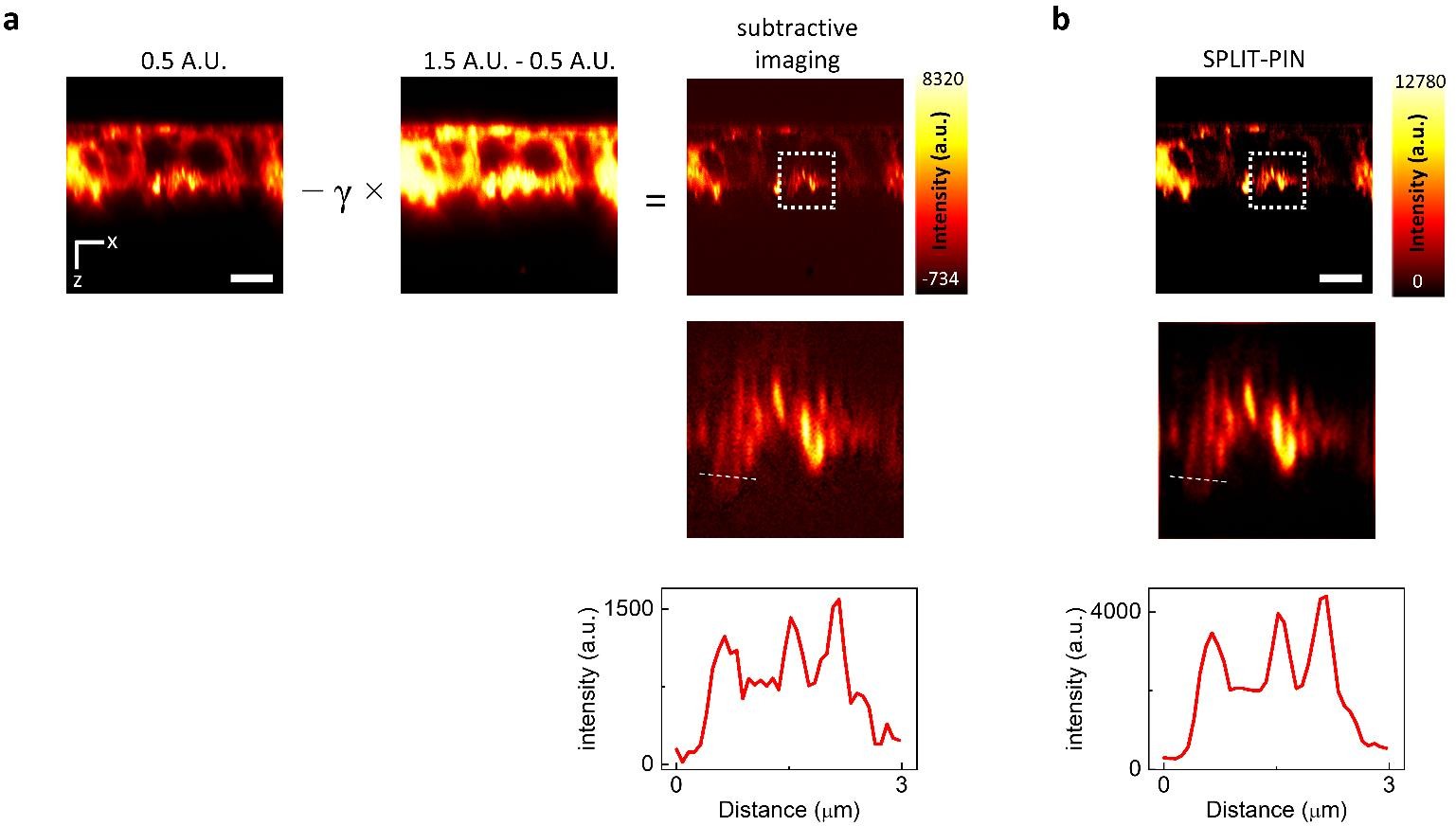


**Figure S1. Comparison between subtractive imaging and SPLIT-PIN imaging.**

a) Subtractive imaging applied to the data of Fig.3. The processed image has been calculated as IPH2-γ(IPH1-IPH2), with γ=0.15, where PH2=0.5AU and PH1=1.5AU. b) The SPLIT-PIN image is shown for comparison. Scale bars 8um.
